# α1-nAchR-Mediated Signaling Through Lipid Raft Is Required for Nicotine-Induced NLRP3 Inflammasome Activation and Nicotine-Accelerated Atherosclerosis

**DOI:** 10.3389/fcell.2021.724699

**Published:** 2021-08-18

**Authors:** Fengqi Duan, Cheng Zeng, Sijun Liu, Jianfeng Gong, Jia Hu, Hongyu Li, Hongmei Tan

**Affiliations:** ^1^Department of Pathophysiology, Zhongshan School of Medicine, Sun Yat-sen University, Guangzhou, China; ^2^Laboratory Animal Center, Sun Yat-sen University, Guangzhou, China; ^3^The Third Affiliated Hospital of Sun Yat-sen University, Guangzhou, China

**Keywords:** lipid raft, nicotine, atherosclerosis, α1-nAChRs, NLRP3, inflammasome

## Abstract

**Background:**

Nicotine exerts direct effects on multiple cell types in the cardiovascular system by associating with its high-affinity nicotinic acetylcholine receptors (nAchRs). Lipid raft is a membrane microdomain that recruits various receptors and signaling molecules for coordinating cellular immune response and many others signaling processes. Here, we aim to identify the essential role of lipid raft in mediating nicotine-triggered inflammatory and nicotine-accelerated atherosclerosis, and to figure out the specific receptor of nicotine-induced Nod-like receptor protein 3 (NLRP3) inflammasome activation in macrophage.

**Methods and Results:**

ApoE^–/–^ mice were fed with a high-fat diet to build atherosclerosis model. Methyl-β-cyclodextrin was used to interrupt intact lipid raft. We confirmed that nicotine triggered NLRP3 inflammasome activation and induced macrophage migration into atherosclerotic plaque, thus accelerated atherosclerosis in apoE^–/–^ mice fed with a high-fat diet. Mechanically, nicotine increased the expression of α1-nAChR and stimulated the accumulation of α1-nAChR in lipid raft, leading to NLRP3 inflammasome activation in macrophage. Conversely, silencing of α1-nAChR in macrophage sufficiently blocked the pro-inflammasome activation effect of nicotine, indicating that α1-nAChR was the specific receptor for nicotine in triggering NLRP3 inflammasome in macrophage. Furthermore, both the destruction of lipid raft by methyl-β-cyclodextrin and the interference of lipid raft clustering by silencing acid sphingomyelinase reversed nicotine-induced NLRP3 inflammasome activation by reducing the accumulation of α1-nAChR in lipid raft in macrophage, suggesting lipid raft–mediated accumulation of α1-nAChR was the key event in regulating the pro-inflammatory effects of nicotine in macrophage. Importantly, nicotine-induced NLRP3 inflammasome activation and macrophage migration into atherosclerotic plaque were reversed by methyl-β-cyclodextrin, making a significant improvement for atherosclerosis in apoE^–/–^ mice fed with a high-fat diet.

**Conclusion:**

α1-nAChR-mediated signaling through lipid raft is required for NLRP3 inflammasome activation and pro-atherosclerotic property of nicotine.

## Introduction

Cigarette smoking now is confirmed to be related to numerous diseases, including cardiovascular disease. Nicotine is the most prominent chemical substance among the 100 components of tobacco and has been considered the major cause of atherosclerosis in recent decades (Lau et al., 2006). Emerging evidence supports the facilitating effect of nicotine on atherosclerosis (Heeschen et al., 2001). Nicotine induces the production of various inflammatory factors, such as interleukin-1β (IL-1β), nuclear factor-κβ, and necrosis factor-α, and NLRP3 inflammasome-mediated pyroptosis was recently considered to be an important pathogenicmechanism on atherosclerosis (Wu et al., 2018). It has been proved that nicotine exerted direct effects on multiple cell types in the cardiovascular system by binding to high-affinity nicotinic acetylcholine receptors (nAchRs) (Lindstrom, 1996; Egleton et al., 2009). The nAchRs consist of five subunits, which are arranged in a barrel-like structure to form a unique channel in the cell membrane. The various combinations of α- and β-subunits lead to functionally different nAchR subtypes with diverse ligand affinity and signaling transduction (Conti-Tronconi et al., 1994; Changeux, 1995). Zhang and colleagues provided evidence that the protein level of the α1 subunit was significantly increased in the aorta of apoE-deficient mice fed with high-fat diet and α1 subunit was mainly localized in macrophages (Lee and Cooke, 2011). However, for macrophage, whether α1-nAchR was the major working subtype for nicotine playing a role in inflammasome activation effect is still unknown.

In atherosclerosis, macrophages play a central role in the initiation, growth, and final rupture of arterial plaque. Various studies based on different purposes have fully proved the vital role of macrophage inflammasome activation in the pathogenesis of atherosclerosis (Tang et al., 2019; Yin et al., 2019; Tian et al., 2020; Wang et al., 2020). NLRP3 inflammasome activation is considered to be a key catalyst for atherogenesis. The activation promoted the production and maturation of IL-1β and IL-18, both of which contribute to atherosclerosis (Libby, 2017). It has been confirmed that NLRP3 inflammasome mainly expressed in cells located in areas of atherosclerotic plaque formation and vascular inflammation (Duewell et al., 2010; Schroder and Tschopp, 2010).

Recent evidence showed lipid raft is an essential component of membrane, which played an important role in immunity effect of macrophage (Schmidt et al., 2020). Lipid rafts are low-density plasma membrane domains composed of dynamic assemblies of cholesterol, lipids carrying saturated acyl chains, such as sphingolipids, and special structural proteins, such as flotillin-1, a 46-kDa marker protein (Nicolau et al., 2006; Rivera-Milla et al., 2006). Lipid raft participated in many cellular processes, which was considered to play an essential role in cell signaling transduction, such as pro-apoptosis and inflammation (Helms and Zurzolo, 2004). The current study intended to investigate whether lipid raft is involved in nicotine-accelerated atherosclerosis and whether lipid raft is the essential platform for the combination of nicotine and α1-nAchRs.

## Materials and Methods

### Animal and Ethics Statement

Wild-type and apoE^–/–^ mice (8–10 weeks; Vital River, Beijing, China) were kept at certified specific pathogen-free facilities around 24°C with a 12-h light/dark cycle and free access to food and water. ApoE^–/–^ mice were randomly divided into high-fat diet (*n* = 8), high-fat diet plus nicotine (*n* = 8), high-fat diet plus nicotine and MβCD co-treated (*n* = 7), and high-fat diet plus MβCD-alone treated (*n* = 5) groups. Wild-type mice fed with normal diet served as the control group (*n* = 7). High-fat diet was obtained from Mediscience Ltd. (MD12017). Mice fed with high-fat diet were drinking the water containing 100 μg/ml nicotine for about 12 weeks to build nicotine-accelerated atherosclerosis. Mice in MβCD co-treated or alone-treated group were subcutaneously injected with MβCD (2 g/kg, 2 times a week) in the last 4 weeks. Our animal experiment was approved by the ethic committees of Sun Yat-sen University, and all procedures were in accordance with the Guide for the Care and Use of Laboratory Animals published by the US National Institutes of Health (NIH Publication No. 85-23, revised 1996). At the end of the experiment, mice were sacrificed by exsanguination after anesthesia with pentobarbital (80 mg/kg body weight). If the mice moved or experienced pain during monitoring, the anesthesia dose was increased up to 100 mg/kg. We assessed the adequacy of anesthesia by monitoring the regular breathing and toe pinch reflex. Blood samples and aortic tissue were collected. The plasma was separated and stored at –80°C until further analysis.

### Histologic Analysis of Aortic Root Plaque

The aortic roots were isolated and then embedded in optimum cutting temperature compound (OCT), and cut into 6- to 8-μm-thick sections. The sections were taken starting from when the three aortic values first appeared and ending from where the aortic values disappeared. All sections were gathered on glass slides and then stained with Oil Red O. The Oil Red O–stained area of the atherosclerotic lesion was imaged under the microscope (Leica DM2500B, Leica, Germany). The percent lesion area was analyzed by Image-Pro Plus 6.0 software.

### Cell Culture and Treatments

Murine RAW264.7 macrophage and human THP-1 monocytic cell line (standard for macrophage) were obtained from American Type Culture Collection (ATCC, Rockville, MD). THP-1 cells were differentiated into macrophage by incubating with Phorbol-12-myristate-13-acetate (PMA, 100 ng/ml) for 24 h. These two cell lines were commonly used to investigate the role of macrophage inflammasome activation in the development of atherosclerosis (Ma et al., 2018; Zhang et al., 2019; Mehta and Dhawan, 2020). The cells were grown in Dulbecco’s Modified Eagle’s Medium (DMEM; Hyclone, South Logan, United States) supplemented with 10% fetal bovine serum (FBS; Gibco, Grand Island, NY, United States) and 1% penicillin/streptomycin (Gibco) with 5% CO_2_ at 37°C. Nicotine and MβCD were purchased from Sigma-Aldrich (St. Louis, MO, United States). When cells reached 60–70%, MβCD (10 mmol/L) was pretreated for 2 h to disrupt lipid raft and then cells were incubated with nicotine for 24 h.

### Lentivirus Injection and Small Interfering RNA (siRNA) Transfection

The lentiviral vectors carrying a short hairpin RNA for α1-nAchR (sh-α1nAchR) or a negative control shRNA (sh-NC) were designed and synthesized by JIKAI Biotechnology Limited Company (Shanghai, China). The sh-α1-nAchR sequences are as follows: sense, 5′-CCGGGCGTGAAGTACATTGCAGAAC CATTTCAAGAGAATGGTCTCTGCAATGTACTTCACGCTT TTG-3′, antisense, 5′-AATTCAAAAAGCGTGAAGTACATTGC AGAGACCATTCTCTTGAAATGGTCTCTGCAATGTACTTC ACGC-3′. The construct was diluted to a total volume of 300 μL containing 1 × 10^8^ TU and administered into RAW264.7.

THP-1 cells were seeded into 6-well plates in DMEM with 10% FBS for 24 h and the next day replaced with fresh culture medium without 1% penicillin/streptomycin. When reaching 50–60%, cells were transfected with 100 nmol/L acid sphingomyelinase (ASM) siRNA named si-ASM or silence negative control RNA named si-NC in catatonic liposome lipofectamine 3000 (Invitrogen, MA, United States) according to the manufacturer’s instructions. After 8 h of transfection, cells were washed and incubated with fresh culture medium with 5 μmol/L nicotine for 24 h. siRNA were designed and purchased from Santa Cruz Biotechnology (Santa Cruz, CA, United States).

### Lipid Raft Extraction

Cells were treated as required for the experiment. The cells were collected with trypsin, washed twice with PBS, and then lysed with 1 ml TNE lysis buffer (containing protease inhibitors and phosphatase inhibitors), and repeatedly pipetted and lysed on ice. The aforementioned solution was centrifuged at 1,000 rpm to remove cell debris. The precipitate was mixed with 2 ml 80% sucrose solution and added it to the ultrafiltration centrifuge tube. Then 4 ml 30% sucrose and 4 ml 5% sucrose solution was added from bottom to top (adding slowly to make it slow from the tube wall). After balancing the sample, it was placed in a Beckman SW40 rotor (Optima L-110xp; Beckman, MA, United States) for centrifugation. After centrifugation at 36,000 rpm for 18 h, the sample was taken out from top to bottom (1 ml per layer, 11 layers in total). After the sample was taken out, total protein in each fraction was determined by western blot.

### Western Blot Analysis

Total protein was collected and concentrations were determined by BCA Protein Assay kit (Thermo Fisher Scientific, MA, United States). Western blot was performed by standard method. Membranes were incubated with the primary antibodies against NLRP3 (Adipogen Life Science, St. Louis, MO, United States), Caspase-1 (Abcam, Cambridge, MA, United States), IL-1β (Abcam), α1-nAchR (Abcam), flotillin-1 (Abcam), and β-actin (Abcam), respectively. Western blot bands were analyzed by chemical touch (Bio-Rad, CA, United States).

### Immunofluorescence Analysis

Immunofluorescence staining method was used according to our previous study (Wang R. et al., 2017). Specific primary antibodies were double stained with frozen aortic root sections for macrophage accumulation analysis, including goat anti-NLRP3 (Abcam) and rat anti-F4-80 (macrophage marker; Abcam) antibodies. Goat anti-NLRP3 (Abcam) and rabbit anti-cleaved caspase-1 antibodies (ImmunoWay, TX, United States) were used for NLRP3 inflammasome activation analysis. Antibodies were double stained with α1-nAchR and CT-XB (lipid raft marker; Abcam) for correlation analysis. The analysis was imaged under a laser scanning confocal microscope (Zeiss, Germany).

### Statistical Analysis

The data were expressed as mean ± SD. All *in vitro* experiments were performed at least three times. GraphPad Prism 8.0 software was used for data analysis. Multiple-group comparison was performed by one-way (ANOVA) test followed by Holm–Šídák *post-hoc* test, and multiple-adjusted *p*-values were displayed for comparisons. *P* < 0.05 was considered statistically significant.

## Results

### α1-nAchR Was the Specific Receptor of Nicotine in Activating NLRP3 Inflammasome in Macrophage

**FIGURE 1:**
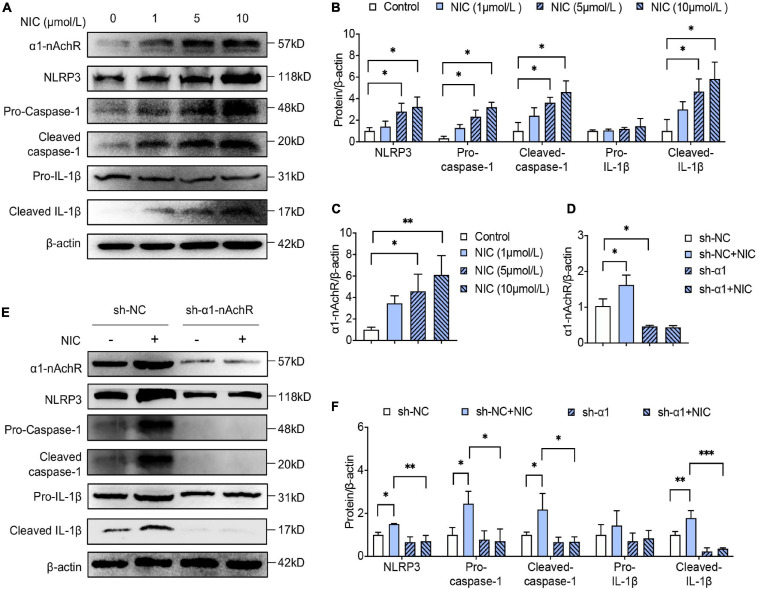
α1-nAchR was the specific receptor of nicotine in NLRP3 inflammasome activation of macrophage. **(A–C)** The protein expression and quantitative analysis of α1-nAchR, NLRP3, and the cleavage of caspase-1 and IL-1β in RAW264.7 cell lines treated with different concentrations of nicotine. **(D–F)** Lentivirus transfection was used to knock down α1-nAchR in macrophage. The protein expression and quantitative analysis of α1-nAchR, NLRP3, and the cleavage of caspase-1 and IL-1β in sh-α1nAchR transfected macrophage. All data are represented as mean ± SD of three independent experiments. ANOVA test followed by Holm–Šídák *post-hoc* test was used for statistical analysis. ^∗^*p* < 0.05, ^∗∗^*p* < 0.01, ^∗∗∗^*p* < 0.001. NIC, nicotine; NLRP3, Nod-like receptor protein 3; sh-NC, short hairpin RNA of negative control; sh-α1nAchR, short hairpin RNA of α1-nAchR.

To elucidate the relationship of α1-nAchR and nicotine in inflammasome activation, RAW264.7 was used for *in vitro* study. On macrophage incubated with nicotine, the expression of α1-nAchR was increased in a dose-dependent manner (Figures 1A,C). Importantly, the expression of NLRP3, the expression and cleavage of caspase-1, and the cleavage of IL-1β were also significantly increased in a dose-dependent manner (Figures 1A,B). These results indicated α1-nAchR was relevant to the activation of NLRP3 inflammasome. As there was no significant difference of α1-nAchR expression and NLRP3 inflammasome activation between macrophage treated with 5 or 10 μmol/L nicotine, we used the concentration of 5 μmol/L for the following research.

To identify whether α1-nAchR was essential to nicotine in activating NLRP3 inflammasome, α1-nAchR was knocked down by lentivirus injection. Using western blot analysis, the protein level of α1-nAchR was reduced by almost 60% after lentiviral transfection of α1-nAchR short hairpin RNA (sh-α1-nAchR) for 48 h compared with lentiviral transfection with negative control RNA (Figures 1D,E). Interestingly, for macrophage transfection with sh-α1-nAchR, nicotine seems to lose its ability in activating NLRP3 inflammasome, evidenced by no significant change in NLRP3 expression and cleavage of caspase-1 and IL-1β. Compared with sh-NC transfected macrophage, we found a rapidly declining expression of NLRP3 and caspase-1 and the decrease of cleavage of caspase-1 and IL-1β in macrophage transfection with sh-α1-nAchR under nicotine environment (Figures 1E,F). All these data indicated that α1-nAchR silence treatment blocked nicotine-induced NLRP3 inflammasome activation, which strongly supported that α1-nAchR was the specific receptor of nicotine in activating NLRP3 inflammasome in macrophage.

### Lipid Raft Disruptor Reversed Nicotine-Induced NLRP3 Inflammasome Activation in Macrophage by Interfering the Aggregation of α1-nAchR Into Lipid Raft

Nicotine exerted direct action by binding to diverse nicotinic acetylcholine receptors in the cell membrane, and lipid rafts, as a specialized membrane domain, were reported to play an essential role in cell signal transduction. Here, we conducted further research to elucidate whether lipid raft mediated the combination of nicotine and α1-nAchR. MβCD, a standard lipid raft destructor, was used to destroy the integrity of lipid raft. The cells were treated with nicotine alone or pre-treated with MβCD.

**FIGURE 2:**
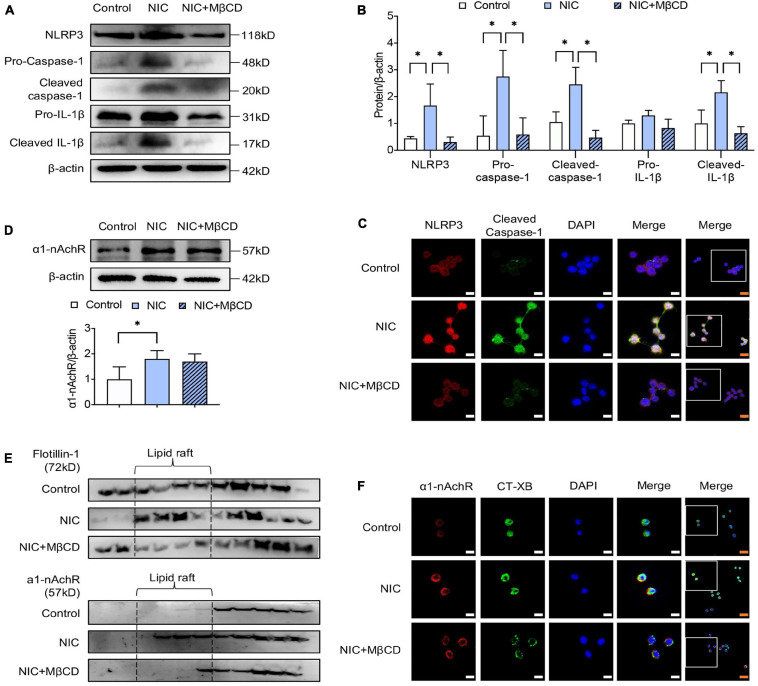
Lipid raft disruptor prevented nicotine-induced NLRP3 inflammasome activation in macrophage by inhibiting a1-nAchR accumulation into lipid raft. MβCD was used as the standard lipid raft disruptor. **(A,B)** The protein expression and quantitative analysis of NLRP3, pro- and cleaved caspase-1 and IL-1β. **(C)** Immunofluorescence showing colocalization of cleaved caspase-1 (green) with NLRP3 (red) in macrophage. **(D)** The protein expression and quantitative analysis of a1-nAchR. **(E)** Immunofluorescence showing colocalization of a1-nAchR (red) and CT-XB (lipid raft marker) (green) in macrophage. **(F)** The protein expression of a1-nAchR and flotillin-1 in lipid raft, *n* = 3. White bars = 5 μm, orange bars = 10 μm. All data are represented as mean ± SD of three independent experiments. ANOVA test followed by Holm–Šídák *post-hoc* test was used for statistical analysis. ^∗^*p* < 0.05. NIC, nicotine; NLRP3, Nod-like receptor protein 3; MβCD, methyl-β-cyclodextrin.

As shown in Figures 2A,B, western blotting analysis showed MβCD treatment attenuated nicotine-induced NLRP3 inflammasome activation, evidenced by decreased expression of NLRP3 and reduced cleavage of caspase-1 and IL-1β. Consistent with the results, double immunofluorescent staining showed MβCD largely reduced nicotine-enhanced NLRP3 and cleaved caspase-1 expression and co-localization on macrophage (Figure 2C).

Interestingly, western blotting showed MβCD treatment had no influence on nicotine-upregulated expression of α1-nAChR (Figure 2D). However, by assessing the protein level of α1-nAChR in lipid raft, we found that the administration of nicotine stimulated α1-nAchR location in lipid raft domain, but MβCD treatment broke the formation of lipid raft and reduced nicotine-induced α1-nAchR accumulation in lipid raft (Figure 2E). To identify the variation of α1-nAchR in lipid raft, immunofluorescent staining was performed. As shown in Figure 2F, nicotine triggered the formation of lipid raft, evidenced by clearly stronger fluorescence intensity (CT-XB, green), but MβCD interrupted nicotine-triggered lipid raft formation, evidenced by the weakened fluorescence intensity. What is more, the co-localization area (yellow) of α1-nAChR (red) and CT-XB (green) on MβCD-treated macrophage was obviously less than nicotine-alone treated group, suggesting MβCD restrained nicotine-induced α1-nAchR accumulation to lipid raft domain, which was consistent with protein analysis. All these results strongly indicated that the destruction of lipid raft by MβCD inhibited nicotine-induced NLRP3 inflammasome activation by suppressing accumulation of α1-nAchR into lipid raft.

**FIGURE 3:**
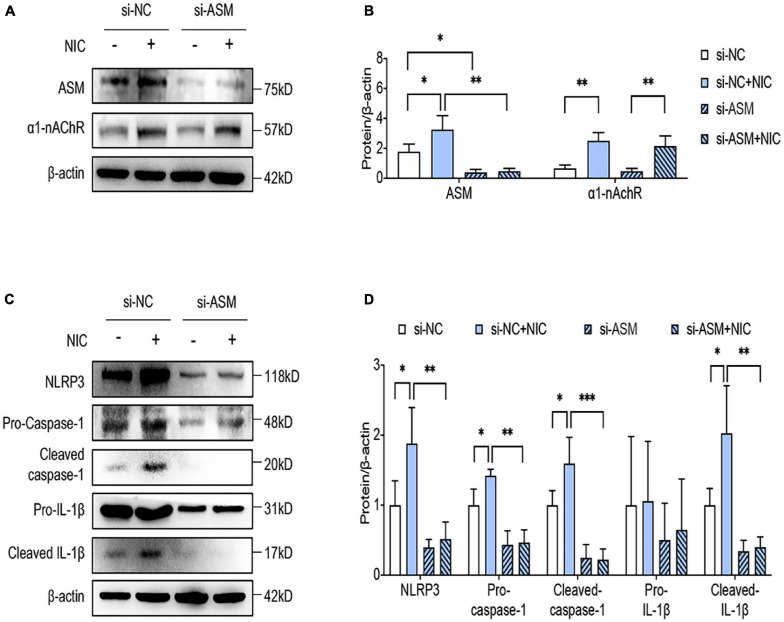
Silencing of ASM blocked nicotine-induced NLRP3 inflammasome activation in macrophage. **(A,B)** The protein expression and quantitative analysis of ASM and a1-nAchR in macrophage. **(C,D)** The protein expression and quantitative analysis of NLRP3, pro- and cleaved caspase-1 and IL-1β in macrophage. All data are represented as mean ± SD of three independent experiments. ANOVA test followed by Holm–Šídák *post-hoc* test was used for statistical analysis. ^∗^*p* < 0.05, ^∗∗^*p* < 0.01, ^∗∗∗^*p* < 0.001. NLRP3, Nod-like receptor protein 3; NIC, nicotine; NLRP3, Nod-like receptor protein 3; ASM, acid sphingomyelinase; si-NC, small interfering RNA of negative control; si-ASM, small interfering RNA of ASM.

ASM activation and translocation produced ceramide, which facilitated individual lipid raft clustering to form large lipid rafts gathering domain. To further confirm the vital role of lipid raft, we knocked down the gene of ASM by siRNA on the THP-1 cell line. By western blot analyses, the silence of ASM was achieved as early as 48 h following siRNA transfection, evidencing by ASM expression in si-ASM treated cell remained approximately 70% lower than the si-NC treated cell (Figures 3A,B). Interestingly, by detecting the protein level of α1-nAchR, we found that the silence of ASM exerted no influence on the expression of α1-nAChR (Figures 3A,B). However, consistent with the administration of MβCD, ASM silencing blocked NLRP3 inflammasome priming and activation in nicotine-treated macrophage, proved by no significant difference in NLRP3 expression and in cleavage of caspase-1 and IL-1β between nicotine-treated or non-treated cells. In addition, comparing si-NC and si-ASM-transfected cells within nicotine administration, a dramatic decline of NLRP3 expression and the cleavage of caspase-1 and IL-1β were presented in si-ASM-transfected macrophage (Figures 3C,D). These data indicated that the interruption of lipid raft by si-ASM transfection inhibited nicotine-induced NLPP3 inflammasome activation in macrophage.

### MβCD Attenuated the Formation of Atherosclerotic Plaque in Nicotine-Treated apoE^–/–^ Mice

**FIGURE 4:**
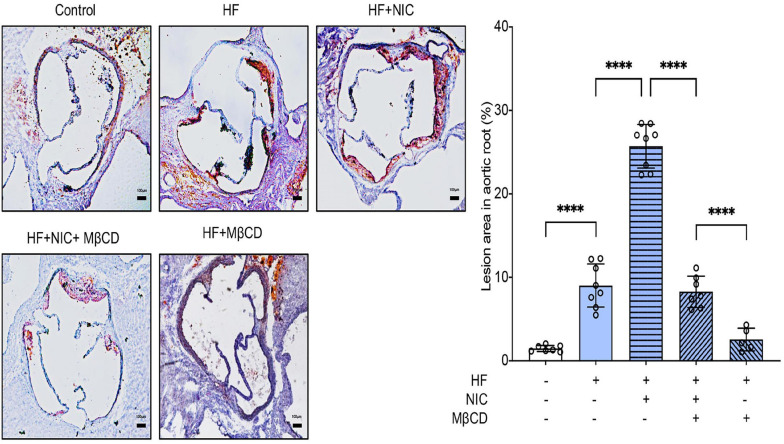
MβCD attenuated the formation of atherosclerotic plaque in nicotine-accelerated atherosclerosis in apoE^–/–^ mice. Mice were randomly divided into control (wild type, normal diet, *n* = 7), high fat (HF) (apoE^–/–^, *n* = 8), nicotine (apoE^–/–^, *n* = 8, high-fat diet), nicotine plus MβCD (apoE^–/–^, *n* = 7, high-fat diet), and MβCD (apoE^–/–^, *n* = 5, high-fat diet) groups. Oil Red O staining showing the atherosclerotic lesions in aortic root and quantitative analysis of atherosclerotic lesion area in the aortic root. Black bars = 100 μm. All data are represented as mean ± SD. ANOVA test followed by Holm–Šídák *post-hoc* test was used for statistical analysis. ^****^p < 0.0001. NIC, nicotine; MβCD, methyl-β-cyclodextrin; HF, high-fat diet.

To assess the effect of lipid raft on nicotine-accelerated atherosclerosis, we fed apoE^–/–^ mice a high-fat diet containing nicotine for 12 weeks, and MβCD was administrated in the last 4 weeks. Wild-type mice fed with normal diet served as the control group. Aortic sinus is most susceptible to the progression of atherosclerosis, so we further explored lipid accumulation in the aortic root. Oil Red O staining were performed. As shown in Figure 4, wild-type mice fed with normal diet exerted almost no formation of atherosclerotic plaque, and apoE^–/–^ mice treated with nicotine alone showed more lipid accumulation compared to non-nicotine treated mice, while the plus administration of MβCD significantly reduced lipid accumulation. These results proved that MβCD attenuated the formation of atherosclerotic plaque in nicotine-accelerated atherosclerosis in apoE^–/–^ mice.

### MβCD Inhibited Nicotine-Induced NLRP3 Inflammasome Activation and Macrophage Recruitment Into Atherosclerotic Lesion in apoE^–/–^ Mice

**FIGURE 5:**
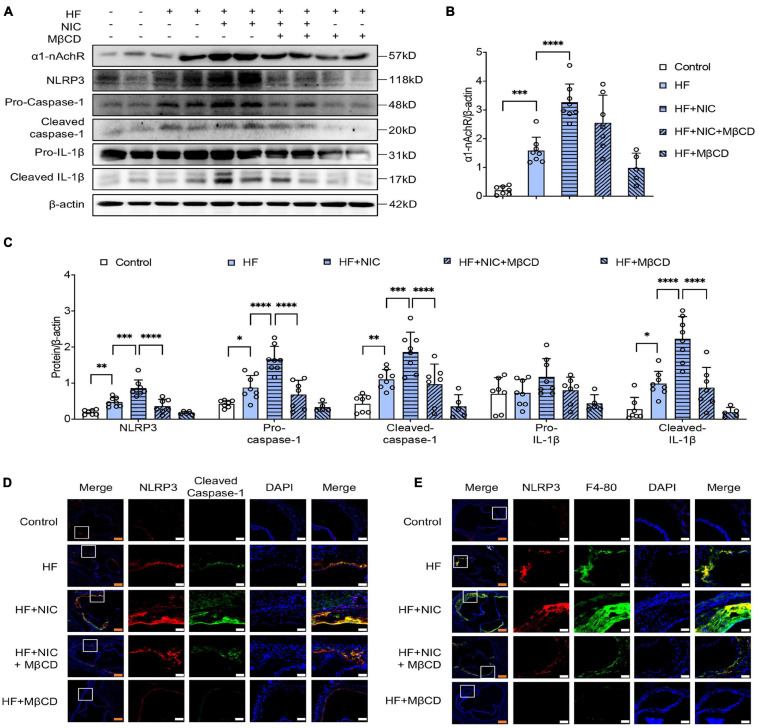
MβCD inhibited nicotine-induced NLRP3 inflammasome activation and macrophage recruitment into atherosclerotic lesion in apoE^–/–^ mice. Mice were randomly divided into control (wild type, normal diet, *n* = 7), high fat (HF) (apoE^–/–^, *n* = 8), nicotine (apoE^–/–^, *n* = 8, high-fat diet), nicotine plus MβCD (apoE^–/–^, *n* = 7, high-fat diet), and MβCD (apoE^–/–^, *n* = 5, high-fat diet) groups. Protein from mice aorta was collected and western blot was performed. **(A–C)** Representative blots and quantitative analysis of a1-nAchR, NLRP3, pro- and cleaved caspase-1 and IL-1β, *n* = 3. **(D,E)** Representative confocal microscopic images showing colocalization of NLRP3 (red) with caspase-1 (green) **(D)**, NLRP3 (red) with F4-80 (green) **(E)** in atherosclerotic lesion. White bars = 25 μm, orange bars = 100 μm. All data are represented as mean ± SD. ANOVA test followed by Holm–Šídák *post-hoc* test was used for statistical analysis. ^∗^*p* < 0.05, ^∗∗^*p* < 0.01, ^∗∗∗^*p* < 0.001, ^****^p < 0.0001. NIC, nicotine; NLRP3, Nod-like receptor protein 3; MβCD, methylcyclodextrin; HF, high-fat diet; F4-80, macrophage marker.

NLRP3 inflammasome activation is an important pathophysiological mechanism in the occurrence and development of atherosclerosis, and it has been shown that nicotine accelerated atherosclerosis mainly through NLRP3 inflammasome activation (Wu et al., 2018). As shown in Figures 5A–C, apoE^–/–^ mice with nicotine administration caused increasing expression of α1-nAchR and NLRP3, and enhanced the cleavage of caspase-1 and IL-1β, which were consistent with the results of other research (Wu et al., 2018). The mice with 4-week administration of MβCD showed significantly downregulated expression of NLRP3, and decreased cleavage of caspase-1 and IL-1β. Meanwhile, contrary to nicotine-alone treated mice, mice co-treated with MβCD and nicotine exerted no difference in the expression of α1-nAchR, which was consistent with our *in vitro* study of macrophage. Double immunostaining of NLRP3 and caspase-1 was performed. The colocalization of NLRP3 (red) and caspase-1 (green) was obviously observed in the aortic section of nicotine-alone treated mice, while mice additionally fed with 4 weeks MβCD presented significantly decreased colocalization area and significantly weakened fluorescence intensity (Figure 5D). All these indicated that MβCD inhibited nicotine-induced NLRP3 inflammasome activation in apoE^–/–^ mice.

The recruitment of macrophage into the injured artery wall was a key contributor to the formation of atherosclerosis plaques. Double staining with anti-NLRP3 and F4-80 antibodies (macrophage marker) was performed. As shown in Figure 5E, wild-type mice as the control group showed almost no macrophage accumulation, and nicotine-alone treated apoE^–/–^ mice had a relatively stronger expression of NLRP3 and F4-80 compared with non–nicotine-treated mice, indicating nicotine stimulated the macrophage recruitment to atherosclerotic plaque. The MβCD and nicotine co-treated mice showed obviously weakened fluorescence intensity of NLRP3 and F4-80 and had less co-localization than nicotine-treated mice. These data suggested that MβCD reversed nicotine-induced macrophage recruitment into atherosclerotic lesion in apoE^–/–^ mice.

## Discussion

The current study demonstrated that nicotine activated NLRP3 inflammasome in macrophage and accelerated atherosclerosis by inducing the accumulation of α1-nAChR in lipid raft. Silencing of α1-nAChR blocked nicotine-induced caspase-1 activation and IL-1β secretion, indicating that α1-nAChR was the functional receptor in nicotine-induced NLRP3 inflammasome activation in macrophage. Destroying the formation of lipid raft by MβCD and interfering the cluster of lipid raft by ASM gene silencing inhibited the accumulation of α1-nAChR into lipid raft, thereby inhibiting nicotine-induced NLRP3 inflammasome activation in macrophage, suggesting lipid raft–mediated α1-nAChR accumulation was the key event in positively regulating nicotine-triggered macrophage inflammation. What is more, MβCD inhibited nicotine-triggered NLRP3 inflammasome activation and reduced nicotine-induced macrophage recruitment to atherosclerotic plaque, thus preventing the progression of atherosclerosis in the aorta of apoE^–/–^ mice fed with high-fat diet. Our study unraveled for the first time that lipid raft–mediated α1-nAChR accumulation was the underlying mechanism for nicotine-induced NLRP3 inflammasome activation in macrophage and for nicotine-accelerated atherosclerosis.

Atherosclerosis is a chronic inflammatory disorder of the arterial wall and the pivotal step in atherosclerosis involves the sub-endothelial accumulation of monocyte-derived macrophages at predisposed sites of endothelial dysfunction and initial lipoprotein retention (Stoger et al., 2012). The apoptosis of macrophage plays a critical role in atherosclerotic lesion development (Martinet et al., 2019). Cigarette smoking encourages the development of atherosclerotic plaques (Ezzati et al., 2005; Al Rifai et al., 2017). Experimental studies have observed that nicotine intake promoted the development of atherosclerosis (Santanam et al., 2012; Wang C. et al., 2017). Consistently, our study showed that nicotine treatment accelerated the formation of atherosclerotic plaques in apoE^–/–^ mice fed with high-fat diet. Our previous study proved NLRP3-mediated macrophage recruitment was responsible for atherosclerotic plaque formation (Tang et al., 2019). Consistent with the previous study, this study showed macrophage recruitment was markedly increased in NLRP3 positive area in the atherosclerotic lesions of nicotine-alone treated mice.

The effect of nicotine on mammal cells is mainly mediated by the specific receptor-nAchR. It has been proved that α1-nAchR played an important role in nicotine-mediated atherosclerosis, evidenced by the silence of α1-nAchR that resulted in restrained atherosclerotic plaque area in apoE^–/–^ mice (Lee and Cooke, 2011). However, the specific mechanism of α1-nAchR in mediating fundamental cell types involved in atherosclerosis, such as macrophage, has not been elucidated. Macrophages are instrumental to atherosclerotic process and contributed to its initiation, progression, and symptomatology. Some studies demonstrated that α1 subunit is expressed on macrophages, and its expression is linked to macrophage chaplain activity and inflammation (Richman and Arnason, 1979). The current study showed that nicotine upregulated the expression of NLRP3, and increased caspase-1 activation and IL-1β secretion, which is related to the increased expression of α1-nAchR both *in vivo* and *in vitro*. Furthermore, the silence of α1-nAchR in macrophage resulted in the inability of nicotine in activating NLRP3 inflammasome. These findings first disclosed that α1-nAchR was the critical receptor of nicotine to exert pro-inflammatory function in macrophage.

Lipid rafts are low-density plasma membrane domains that are involved in many biological events, such as trafficking, synthetic traffic, and cell signaling (Mineo et al., 1996; Pike, 2003; Helms and Zurzolo, 2004). Lipid rafts provide a solid platform for the assembly of macromolecular complexes in the membrane. The enrichment and functional properties of lipid rafts changed rapidly according to changes in metabolic conditions (Sviridov et al., 2020). The dysregulation of lipid rafts played a key role in the pathogenesis of inflammatory, infectious diseases and cancer. One study demonstrated that LPS stimulated macrophage inflammation by encouraging the recruitment of TLR4 and MyD88 into lipid raft (Chen et al., 2020). Our study demonstrated that nicotine induced the activation of NLRP3 inflammasome in macrophage by prompting the recruitment of α1-nAchR in lipid raft.

Currently, there are two mechanisms in regulating lipid rafts’ dynamic remodeling. One is dependent on the structural lipids of cholesterol and sphingolipids of lipid rafts, the important lipid components of lipid rafts (Cremesti et al., 2002; Zhuang et al., 2005). Using MβCD to deplete cholesterol in lipid rafts was a classic method of breaking down lipid rafts. This method significantly reduces all signal transduction mediated by lipid rafts (Yang et al., 2018). In this context, our study demonstrated that the administration of MβCD inhibited the trafficking of α1-nAchR into lipid raft, thus inhibiting nicotine-triggered NLRP3 inflammasome activation in macrophage. Meanwhile, significant inhibition of nicotine-accelerated atherosclerotic plaque formation was shown in mice fed with MβCD, suggesting α1-nAchR/lipid raft-mediated NLRP3 inflammasome signal may be the potential mechanism for the activation of macrophage and the progression of atherosclerosis.

As we know, individual lipid rafts only contain a subset of all available raft proteins, so raft clustering is important for transmembrane signaling through its amplification (Zhang and Li, 2010). Ceramide, mainly generated by SMase pathway, is the crucial binder for individual lipid raft clustering. The biophysical properties of ceramide predict a tight interaction of ceramide molecular with each other, resulting in the formation of stable and tightly packed ceramide-enriched membrane microdomains (Jia et al., 2008). ASM was considered to be the major enzyme among SMase responsible for the formation of ceramide-enriched lipid raft (Zhang et al., 2008; Bao et al., 2010). To further verify the key role of lipid raft, we silenced ASM on THP-1 cell lines to reduce ceramide production in membrane, thus individual lipid raft could not cluster to form a large platform. Consistent with MβCD treatment on RAW264.7, the silence of ASM blocked nicotine-induced NLRP3 inflammasome activation. These results strongly demonstrated lipid raft–mediated accumulation of α1-nAChR was responsible for nicotine-induced NLRP3 inflammasome activation in macrophage.

This study demonstrates for the first time that α1-nAChR is the specific receptor for nicotine in activating NLRP3 inflammasomes in macrophage, and the accumulation of α1-nAChR in lipid raft is the key event for pro-inflammatory effect of nicotine. Lipid raft destructor MβCD inhibits nicotine-induced NLRP3 inflammasome activation in macrophage and alleviates nicotine-accelerated atherosclerosis. Our study highlights the importance of lipid raft in promoting inflammation and provides new insight into the therapeutic effect of α1-nAChR and lipid raft on nicotine-accelerated atherosclerosis.

## Data Availability Statement

The original contributions presented in the study are included in the article/supplementary material, further inquiries can be directed to the corresponding author/s.

## Ethics Statement

The animal study was reviewed and approved by the Sun Yat-sen University.

## Author Contributions

FD and CZ designed and performed the research. FD collected and analyzed the results and prepared the original draft preparation. CZ, SL, JG, and JH performed the experiments. HL helped to establish the animal model. HT obtained funding for the project, initiated and supervised the project, designed the research, analyzed and interpreted the results, and revised the manuscript. All authors reviewed the results and approved the final version of the article.

## Conflict of Interest

The authors declare that the research was conducted in the absence of any commercial or financial relationships that could be construed as a potential conflict of interest.

## Publisher’s Note

All claims expressed in this article are solely those of the authors and do not necessarily represent those of their affiliated organizations, or those of the publisher, the editors and the reviewers. Any product that may be evaluated in this article, or claim that may be made by its manufacturer, is not guaranteed or endorsed by the publisher.
